# Beam geometry optimization and convergence mode evaluation for knowledge‐based planning of hippocampal avoidance whole‐brain radiotherapy: A cross‐platform study with quantitative scorecard assessment

**DOI:** 10.1002/acm2.70771

**Published:** 2026-08-25

**Authors:** Anh Lam, Kirk Luca, Mandeep Kaur, Keyur D. Shah, Julia Seng, Kathryn Benner, Matthew Thomas, Hui‐Kuo Shu, Xiaofeng Yang, Chih‐Wei Chang, Justin Roper, Jie Ding

**Affiliations:** ^1^ Department of Radiation Oncology Emory University Atlanta Georgia USA; ^2^ Medical Dosimetry Program Southern Illinois University Carbondale Illinois USA; ^3^ Department of Radiation and Cellular Oncology University of Chicago Chicago Illinois USA

**Keywords:** arc configuration, beam geometry, convergence mode, hippocampal avoidance whole‐brain radiotherapy, volumetric modulated arc therapy

## Abstract

**Background:**

Hippocampal avoidance whole‐brain radiotherapy (HA‐WBRT) is used to treat brain metastases while preserving cognitive function by sparing the hippocampi. Volumetric modulated arc therapy (VMAT) is commonly used due to its ability to generate conformal dose distributions. However, treatment planning remains challenging due to the need for sharp dose fall‐off near centrally located hippocampi without compromising whole‐brain coverage.

**Purpose:**

This study evaluated the dosimetric impact of a four‐arc hybrid geometry (4A‐HG) and convergence mode settings for HA‐WBRT using a knowledge‐based planning (KBP) workflow across two linac platforms.

**Methods:**

Twenty HA‐WBRT patients prescribed 30 Gy in 10 fractions were retrospectively studied. Plans were generated using a KBP model on two linac platforms with different collimation systems: Halcyon and Edge. Four arc configurations were evaluated, including three open‐field geometries with different numbers of arcs (2A, 3A, and 4A) and 4A‐HG. The 4A‐HG consists of two open‐field arcs and two partial‐field arcs with superior‐inferior field restriction near the hippocampal region, which allows improved dose modulation near the hippocampus to potentially enhance hippocampal sparing. Jaw tracking was enabled for all Edge plans. Each arc configuration was combined with three convergence modes (“Off,” “On,” and “Extended”) on both linac platforms, yielding 24 plans per patient. Plans were normalized so that 95% of the planning target volume (PTV) received 100% of prescription dose. Plan quality was evaluated using Radiation Therapy Oncology Group (RTOG) 0933 metrics, as well as by a dosimetric scorecard incorporating PTV D100% and the Paddick conformity index (PCI). Statistical comparisons were performed using Friedman tests with Holm correction. Patient‐specific quality assurance (QA) was performed for the five highest‐monitor‐unit (MU) plans on each platform.

**Results:**

The proposed 4A‐HG consistently improved target coverage and hippocampal sparing compared with conventional open‐field arc configurations. Notably, PTV D100% was significantly improved with 4A‐HG on the Edge with the “On” and “Extended” convergence modes (all *p* < 0.05 compared with other arc configurations). For example, with the “On” convergence mode, mean PTV D100% across 20 patients increased from 19.433 Gy with 2A to 20.011 Gy with 4A‐HG (*p* = 0.009). The 4A‐HG also achieved the highest mean PCI using the “Extended” mode (0.940 on Halcyon, 0.944 on Edge). Hippocampus Dmax was significantly reduced with 4A‐HG on Halcyon across all modes (*p* < 0.01) and on Edge for “On” and “Extended” modes (*p* < 0.05). On Halcyon, the mean hippocampus Dmax decreased from 14.446 Gy to 13.662 Gy when changing the arc configuration from 2A to 4A‐HG using the “Extended” mode, with a similar reduction observed on Edge (12.945 Gy to 12.566 Gy). More extensive convergence mode settings further improved plan quality, with the highest mean score (157/184) achieved on Edge using 4A‐HG under the “Extended” mode. These improvements were associated with increased MUs; however, all QA results met institutional criteria.

**Conclusions:**

The 4A‐HG arc configuration and the more extensive convergence mode settings improved plan quality for HA‐WBRT within a KBP workflow across two linac platforms. These findings support the clinical implementation of the proposed planning strategies for VMAT‐based HA‐WBRT.

## 1 INTRODUCTION

Brain metastases are the most common intracranial tumors in adults and occur in up to 40% of patients with cancer.^1^ Although the incidence is difficult to determine accurately, estimates suggest that 100,000–200,000 patients develop brain metastases annually in the United States, with increasing rates reported in more recent clinical and autopsy studies.^1^ Historically, whole‐brain radiotherapy (WBRT) has served as the standard of care in managing brain metastases.^2^ WBRT is conventionally delivered using two opposed lateral fields, with typical fractionations of 30 Gy in 10 fractions or 20 Gy in 5 fractions.^2^ However, conventional WBRT is associated with neurocognitive toxicity, potentially due to irradiation of the hippocampus, which plays an essential role in memory function.^3^ A previous study demonstrated that an equivalent dose in 2‐Gy fractions greater than 7.3 Gy to 40% of the bilateral hippocampi predicted persistent memory impairment on long‐term follow‐up.^3^ This dose‐response relationship has motivated the development of planning approaches designed to reduce hippocampal irradiation while maintaining whole‐brain coverage.

One approach is volumetric modulated arc therapy (VMAT) based hippocampal‐avoidance WBRT (HA‐WBRT), which enables a highly conformal dose distribution that spares the hippocampi compared with conventional opposed‐field WBRT. Following the clinical trials of Radiation Therapy Oncology Group (RTOG) 0933 and NRG Oncology CC001,^4^, ^5^ HA‐WBRT has been increasingly adopted in clinical practice to help preserve cognitive functions in patients with brain metastases. However, HA‐WBRT treatment planning is challenging because the hippocampi are intricately shaped and centrally located within the brain. Therefore, substantial planning time and expertise are required to achieve the steep dose gradients required for hippocampal sparing while maintaining adequate whole‐brain coverage. Failure to maintain adequate whole‐brain coverage, particularly adjacent to the hippocampi, may increase recurrence risk from occult metastases, while insufficient hippocampal sparing may reduce the intended neurocognitive benefit.^6^

Previous studies have investigated various VMAT planning strategies to improve plan quality for HA‐WBRT. Several studies proposed non‐coplanar irradiation and reported improvements in dose homogeneity and conformity.^7^ However, this approach may increase the risk of intrafraction motion as well as delivery complexity and efficiency.^8^ Other investigations have focused on coplanar arc techniques, evaluating the dosimetric impact of different planning strategies such as varying the number of arcs^9^, ^10^ and reducing field size.^10^, ^11^ Dosimetric performance has also been evaluated across different treatment platforms, including C‐arm and O‐ring linear accelerators (linacs) and helical tomotherapy.^10^, ^12^ In addition, machine learning‐driven knowledge‐based planning (KBP), has been explored in recent years for HA‐WBRT.^9^, ^13^ KBP is a semi‐automated planning approach that builds dose‐volume histogram (DVH) models from prior high‐quality plans and generates dose objectives for patients with similar anatomy, which can potentially improve consistency in plan quality, reduce inter‐operator variability and enhance the efficiency of the planning process.^14^

Although previous studies have aimed at improving HA‐WBRT treatment planning, the dosimetric impact of different arc designs, collimator settings, and optimization parameters when using KBP on modern linacs has not been fully investigated. Previous techniques, such as split‐arc partial‐field VMAT, used restricted field sizes and specific jaw settings to better protect the hippocampus from low‐dose exposure.^11^ However, integrating such approaches into a standardized KBP workflow requires further study. In this study, we introduce a Four‐Arc Hybrid Geometry (4A‐HG) geometry that uses two open‐field arcs for global target coverage and two partial‐field arcs that limit the field opening at the superior and inferior boundaries of the hippocampi. This superior‐inferior field split restricts field sizes and therefore allows for improved dose modulation near the hippocampal region by minimizing the effects of island blocking, which potentially enables additional dose sparing without compromising whole‐brain coverage. In addition, while the effect of convergence mode settings in the Eclipse treatment planning system (Varian Medical Systems, USA) has been evaluated for other treatment sites,^15^, ^16^, ^17^, ^18^ their potential to further improve HA‐WBRT plan quality remains underexplored. Furthermore, it is important to evaluate how these beam designs and optimization settings perform across linacs with different multileaf collimator (MLC) architectures.

The goal of this study was to investigate the dosimetric impact of our proposed beam geometry 4A‐HG for HA‐WBRT compared with conventional open‐field geometry using varying numbers of arcs, on both an O‐ring linac (Varian Halcyon) and a C‐arm linac (Varian Edge), with planning facilitated by KBP. Plan quality was evaluated not only according to the dosimetric constraints specified in RTOG 0933 but also using a custom‐designed dosimetric scorecard to provide additional quantitative assessment. By systematically comparing plan quality across distinct arc configurations and convergence modes on two treatment delivery platforms, this work aims to identify a practical and standardized planning workflow that balances whole‐brain coverage and hippocampal sparing.

## 2 METHODS

### 2.1 Patient selection

This study was approved by the Institutional Review Board. Twenty HA‐WBRT patients were randomly selected for this retrospective study. All plans used a prescription of 30 Gy in 10 fractions.

### 2.2 RTOG 0933 contouring and planning guidelines

All contours were generated in strict accordance with RTOG 0933 guidelines.^4^ The bilateral hippocampi were contoured by a radiation oncologist on a T1‐weighted MRI and transferred to the simulation CT using rigid MRI‐to‐CT fusion. A hippocampal avoidance planning risk volume (PRV) was then created by applying a 5‐mm expansion to each hippocampus to guide optimization and limit hippocampal dose. The whole‐brain planning target volume (PTV) was subsequently segmented and cropped from the hippocampal PRV with no additional margin to generate the primary treatment target. The hippocampus contours, including the PRV, were converted to high‐resolution structures to improve dose calculation accuracy in Eclipse. Additional organs‐at‐risk (OARs) contoured for treatment planning and dose evaluation included the optic nerves and optic chiasm. Dosimetric constraints specified in RTOG 0933 are listed in Table S1.

### 2.3 MLC configurations of the treatment delivery systems

Varian Halcyon is an O‐ring linac configured with a stacked and staggered dual‐layer MLC system and no conventional collimator jaws. Each MLC leaf has a projected width of 10 mm at isocenter but provides an effective spatial resolution of 5 mm because of the staggered dual‐layer design. The proximal and distal MLC layers consist of 29 and 28 leaf pairs, respectively, allowing a maximum field size of 28 × 28 cm^2^ at isocenter, with MLC leaves capable of traveling across the full 28 cm field width.

Varian Edge is a C‐arm linac incorporating two conventional collimator jaws and a tertiary high‐definition MLC (HD120 MLC) system positioned below the jaws. The HD120 MLC contains 60 leaf pairs, including 32 central pairs with a projected leaf width of 2.5 mm at isocenter and the remaining 28 pairs with a projected width of 5 mm at isocenter, creating a maximum field length of 22 cm. The maximum VMAT field size is 32 × 22 cm^2^ at isocenter. The maximum distance between the most extended and most retracted leaf positions is 15 cm per MLC bank.

Figure S1 shows the beam's‐eye view (BEV) of the MLC configurations at an example VMAT control point for the two treatment machines.

### 2.4 Treatment planning and beam geometry

All treatment plans were generated in Eclipse v16.1 using the Photon Optimizer with an in‐house KBP RapidPlan model, incorporating a monitor unit (MU) objective (minimum of 1000, maximum of 2000, strength of 50). No user interaction was required beyond structure matching to generate RapidPlan objectives. The in‐house RapidPlan model was developed using 25 high‐quality clinical treatment plans, validated on an independent cohort of 8 patients, and approved for clinical use. The validation cohort was completely independent of the training dataset. The model details, including objective template and custom normal tissue objective settings, are provided in Tables S2 and S3. Plans were created using 6‐MV flattening filter‐free (6X‐FFF) beams on the two delivery platforms: Halcyon (maximum dose rate 800 MU/min) and Edge (maximum dose rate 1400 MU/min). Dose calculations were performed with the Analytical Anisotropic Algorithm (AAA) using a 1 mm dose calculation grid for all plans evaluated in this study.

**FIGURE 1:**
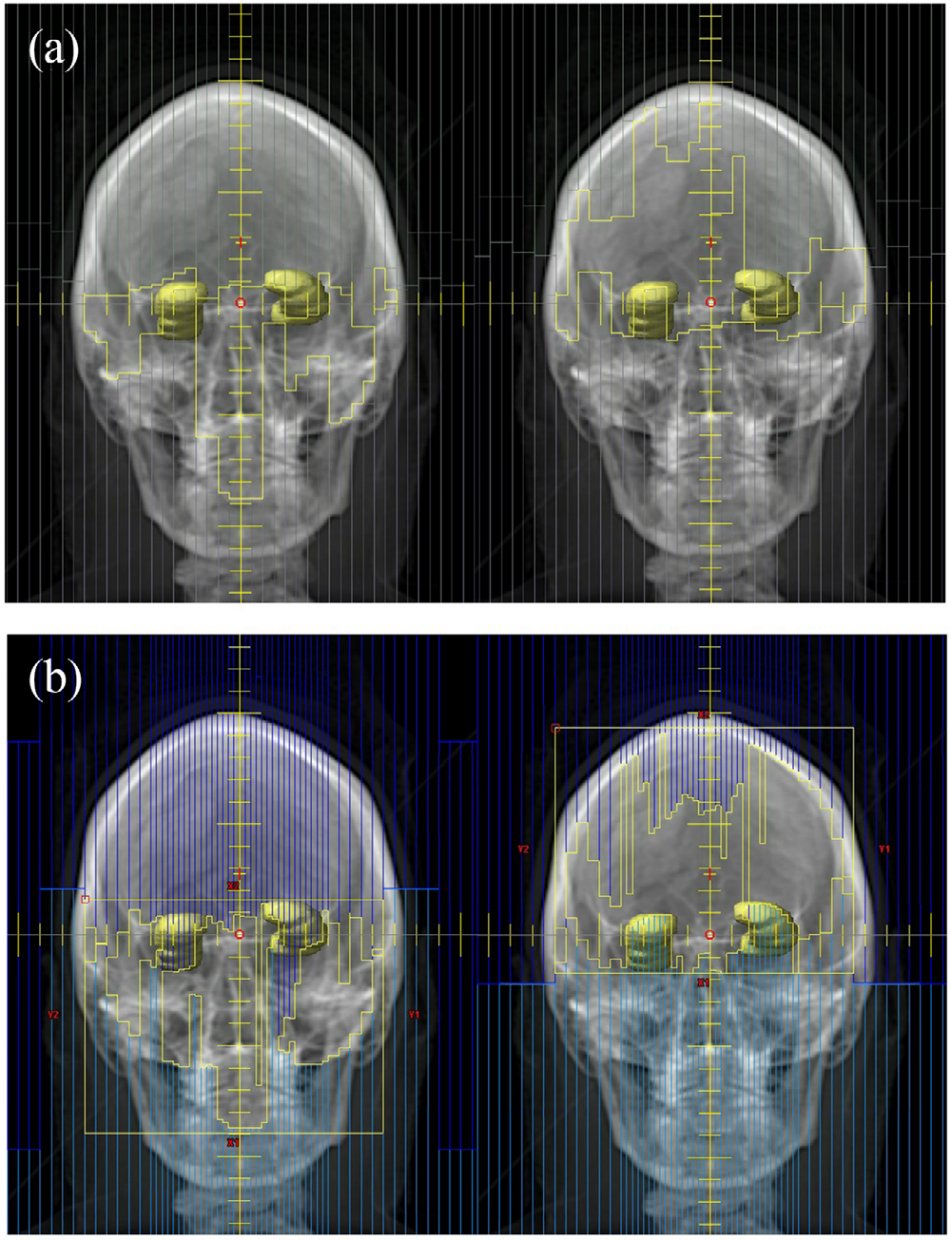
Example beam's‐eye views (BEVs) of the partial‐field arcs in 4A‐HG arc configuration illustrating field truncation near the hippocampus PRV (yellow) to improve sparing. (A) Halcyon: inferior and superior field extents limited at the edge of the hippocampus PTV using the “limit MLC” feature; (B) Edge: inferior and superior field extents limited at the edge of the hippocampus PTV using jaws.

Four arc configurations were evaluated. The first three configurations consisted of 2‐, 3‐, and 4‐arc full‐field VMAT plans with open apertures that allowed the PTV to be fully encompassed throughout each arc. Collimator angles of 315° and 45° were used for all full‐field VMAT plans. These complementary collimator angles provide different MLC orientations between the arcs and have also been used in a previously reported HA‐WBRT planning technique.^9^ The fourth configuration (4A‐HG) used two full‐field arcs (collimator angles of 315° and 45°) for global target coverage and two collimator‐restricted arcs (collimator angle of 90°) with superior and inferior MLC/jaw borders defined near the hippocampal region. The 90° collimator angle, together with the restricted aperture, allows the MLC leaves to move parallel to the hippocampal avoidance region, facilitating hippocampal sparing while maintaining an open aperture over the remainder of the target (Figure 1). On the Varian Halcyon, a jawless treatment platform, the “limit MLC” feature in the optimization workspace was used to restrict the superior and inferior field extent near the hippocampus PRV (Figure 1A). On the Varian Edge, the superior and inferior field borders were truncated near the hippocampus PRV using the X1 or X2 jaws (Figure 1B). Jaw tracking was enabled for all Edge plans. Table S4 summarizes the arc configurations used in this study.

For each arc configuration, plans were optimized using three convergence mode settings (“Off”, “On”, and “Extended”) in Eclipse. The convergence mode controls the maximum number of optimization iterations and the convergence criteria for the cost function. The “On” and “Extended” options offer additional iterations and apply more stringent convergence criteria than the “Off” setting, with the “Extended” mode allowing the most extensive optimization. The additional optimization of the planning objectives can potentially enhance dosimetric performance at the expense of longer optimization times, as reported in previous studies on other treatment sites.^15^, ^16^, ^17^, ^18^

**FIGURE 2:**
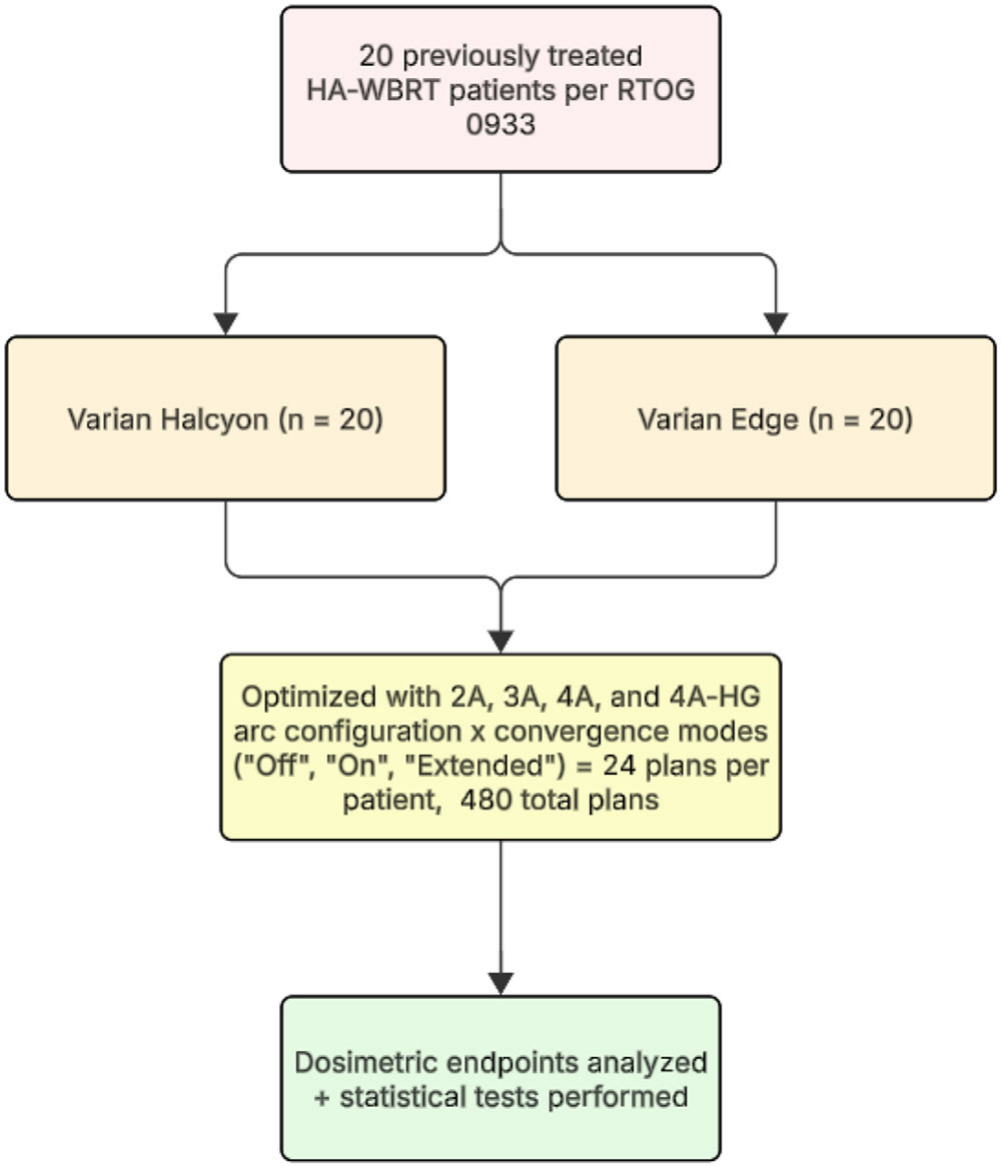
A flow chart showing the workflow for this study.

All plans were normalized such that 30 Gy covered 95% of the PTV. Evaluating all combinations (4 arc configurations × 3 convergence modes) yielded 12 plans per linac platform, for a total of 24 plans per patient. Figure 2 shows the workflow for this study.

### 2.5 Plan evaluation

**FIGURE 3:**
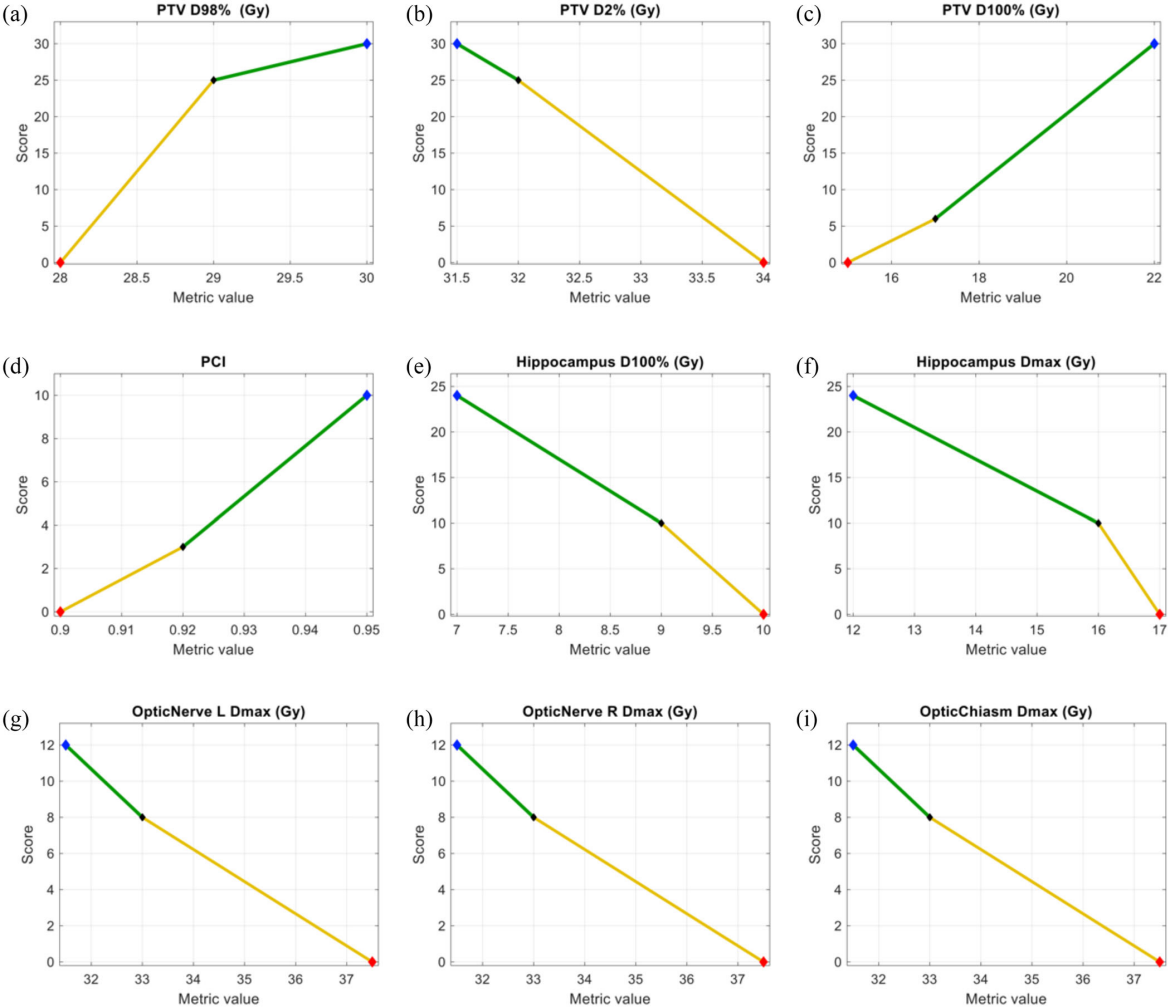
Piecewise linear scoring functions for each metric in the custom dosimetric scorecard, defined by lower, breakpoint, and upper thresholds, with scores between thresholds determined by linear interpolation.

Plan evaluation was performed by assessing target coverage and OAR sparing using dosimetric metrics in accordance with RTOG 0933 (Table S1), as well as a custom‐designed dosimetric scorecard to assist with overall plan quality review. The scorecard is a useful tool to provide quantitative and comprehensive evaluation by integrating multiple dosimetric metrics through piecewise linear score functions with different weights into a single scoring scheme. The scorecard metrics were developed based on the RTOG 0933 criteria and supplemented with additional clinically relevant endpoints routinely used in our clinical practice, including the minimum dose received by the PTV (PTV D100%) and Paddick conformity index (PCI).^19^ It is noted that PTV D100% is an important metric to ensure the whole‐brain receives as much target coverage as possible, and it also reflects the balance between target coverage and dose fall‐off near the hippocampus. This dosimetric goal reflects our institutional physician preference during clinical plan review and has rarely been reported in previous studies on HA‐WBRT treatment planning. PCI was calculated as described by Paddick,^19^ which accounts for the spatial overlap between the prescription isodose volume and the target volume. For PCI, a value of 1 indicates perfect conformity; values closer to 1 are preferred. Each dosimetric endpoint was assigned a weight according to its relative importance in treatment planning, and the piecewise linear scoring functions were designed to reflect the clinical relevance of each endpoint. The piecewise linear scoring functions for the custom dosimetric scorecard are provided in Figure 3, with the detailed scoring design for each dosimetric endpoint shown in Table S5, and a maximum total score of 184.

The effect of arc configuration (2A, 3A, 4A, and 4A‐HG) and convergence mode (“Off”, “On”, and “Extended”) were compared separately using the Friedman test, with Holm correction applied to account for multiple pairwise comparisons. Holm‐adjusted *p* < 0.05 was considered statistically significant.

Total MUs were recorded for all plans, with patient‐specific quality assurance (QA) performed for the five plans with the highest total MUs on each of the two treatment machines to evaluate plan deliverability and dosimetric accuracy. Measurements were acquired using electronic portal imaging devices (EPIDs), with gamma passing rates evaluated on a field‐by‐field basis using criteria of 3% dose difference and 2 mm distance‐to‐agreement with a 10% dose threshold and global normalization.

## 3 RESULTS

### 3.1 Spatial visualization of hippocampal sparing

**FIGURE 4:**
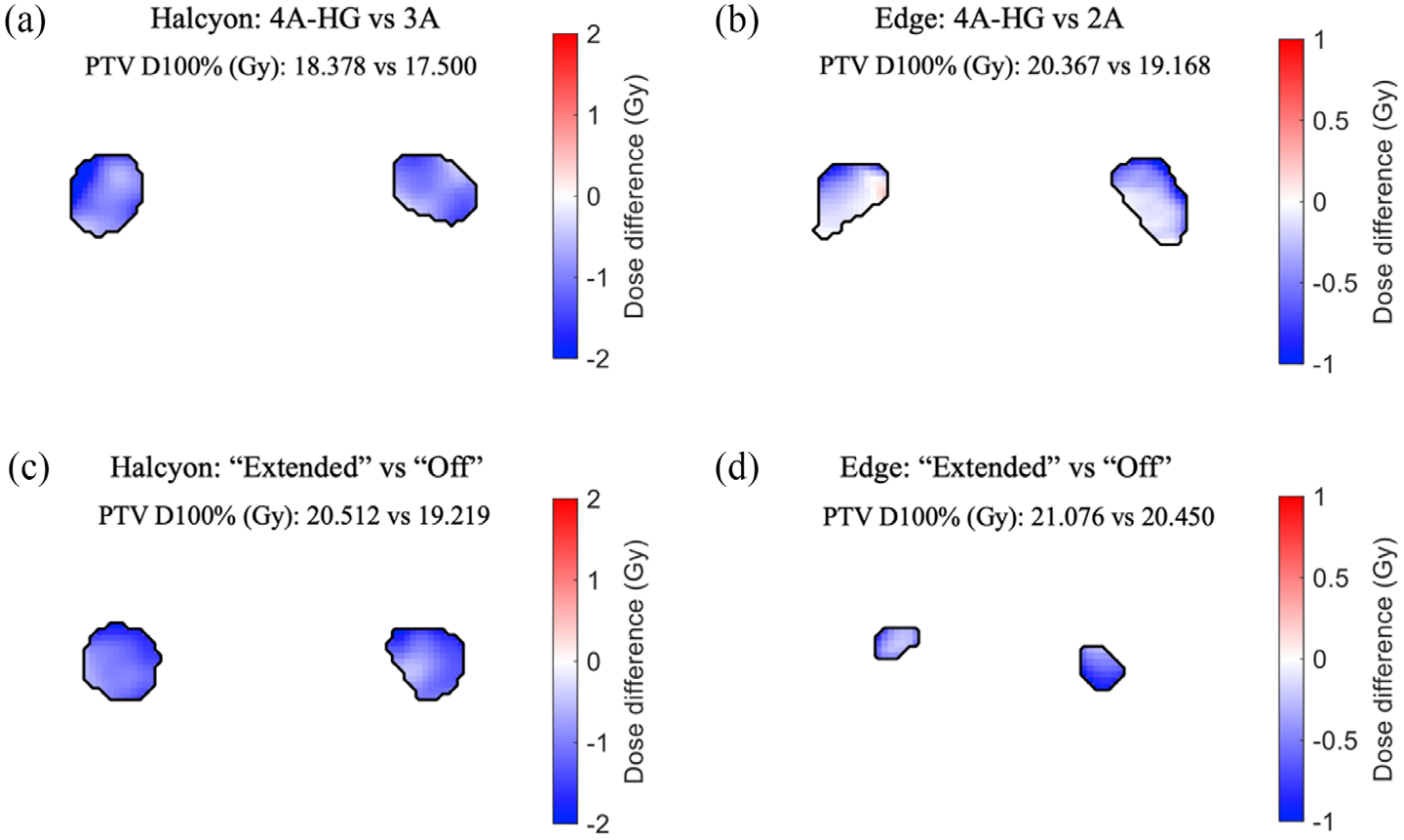
Representative dose difference maps for selected example cases on Halcyon and Edge illustrating hippocampal sparing achieved with the proposed 4A‐HG beam geometry (A and B) and “Extended” convergence mode (C and D). The corresponding PTV D100% values are listed to demonstrate that target coverage improved alongside the enhanced hippocampal sparing.

Representative dose difference maps are shown in Figure 4 to illustrate the spatial dosimetric effects of the proposed 4A‐HG beam geometry and convergence mode strategies in selected example cases. Compared with conventional open‐field arc geometries, the proposed 4A‐HG approach demonstrated reduced dose in the hippocampal avoidance regions on both Halcyon and Edge platforms while further improving the target coverage, as reflected by the improved PTV D100% values. Similarly, the “Extended” convergence mode further improved hippocampal sparing and target coverage compared with the “Off” mode on both platforms. These spatial dosimetric trends were consistent with the quantitative results presented in the following section.

### 3.2 Dosimetric metrics

All dosimetric goals specified in RTOG 0933 were evaluated along with PTV D100% and PCI. Among the 480 plans generated, all plans achieved the RTOG 0933 per‐protocol goals for PTV D98%, PTV D2%, optical nerves and chiasm Dmax, and met the acceptable variation for hippocampus D100%, as listed in Table 1. For Hippocampus Dmax, 6 of the 480 plans exceeded the unacceptable threshold (> 17Gy), all of which used the “Off” convergence mode on Halcyon (1 with 2A, 2 with 3A, 2 with 4A, and 1 with 4A‐HG).

**TABLE 1:** Selected dosimetric metrics, scorecard results, and MUs for plans across arc configurations and convergence modes on Halcyon and Edge. Values are reported as mean ± standard deviation for 20 patients. Bold values indicate the best dosimetric performance among arc configurations within each convergence mode.

		Halcyon			Edge		
Metrics	Arc configuration	Convergence mode “Off”	Convergence mode “On”	Convergence mode “Extended”	Convergence mode “Off”	Convergence mode “On”	Convergence mode “Extended”
PTV D100% (Gy)	2A	18.482 ± 0.906	18.672 ± 1.201	18.948 ± 1.121	19.206 ± 1.265	19.433 ± 1.224	19.613 ± 1.254
	3A	18.470 ± 1.280	18.668 ± 1.111	18.880 ± 1.232	19.082 ± 1.267	19.343 ± 1.170	19.402 ± 1.212
	4A	18.384 ± 1.230	18.492 ± 1.169	18.787 ± 1.107	19.005 ± 1.212	19.432 ± 1.174	19.498 ± 1.222
	4A‐HG	**18.740 ± 1.108**	**19.028 ± 1.116**	**19.416 ± 1.427**	**19.507 ± 1.022**	**20.011 ± 1.202**	**20.170 ± 1.208**
PCI	2A	0.913 ± 0.007	0.921 ± 0.006	0.923 ± 0.007	0.928 ± 0.006	0.934 ± 0.005	0.936 ± 0.005
	3A	0.921 ± 0.007	0.927 ± 0.007	0.931 ± 0.006	0.935 ± 0.006	0.939 ± 0.005	0.942 ± 0.004
	4A	0.924 ± 0.007	0.930 ± 0.006	0.934 ± 0.005	**0.937 ± 0.005**	**0.941 ± 0.004**	0.943 ± 0.004
	4A‐HG	**0.929 ± 0.007**	**0.933 ± 0.009**	**0.940 ± 0.005**	**0.937 ± 0.005**	**0.941 ± 0.004**	**0.944 ± 0.004**
Hippocampus Dmax (Gy)	2A	15.545 ± 0.885	14.863 ± 0.815	14.446 ± 0.896	13.577 ± 0.762	13.157 ± 0.685	12.945 ± 0.661
	3A	15.623 ± 1.028	14.913 ± 1.015	14.392 ± 0.962	13.711 ± 0.870	13.150 ± 0.726	12.834 ± 0.673
	4A	15.769 ± 0.950	14.916 ± 0.885	14.323 ± 0.921	13.574 ± 0.759	13.054 ± 0.639	12.744 ± 0.521
	4A‐HG	**15.144 ± 0.942**	**14.370 ± 0.838**	**13.662 ± 0.813**	**13.508 ± 0.796**	**12.863 ± 0.690**	**12.566 ± 0.705**
Scorecard	2A	98.31 ± 9.63	110.83 ± 11.00	119.57 ± 11.39	130.94 ± 11.17	140.90 ± 9.53	145.36 ± 9.68
	3A	103.17 ± 12.39	116.87 ± 11.63	126.41 ± 11.23	136.30 ± 11.13	145.65 ± 9.10	150.86 ± 9.45
	4A	103.65 ± 12.65	118.61 ± 10.59	129.40 ± 10.68	138.08 ± 11.17	148.52 ± 8.88	153.87 ± 8.55
	4A‐HG	**114.32 ± 12.08**	**128.90 ± 9.24**	**142.34 ± 10.55**	**138.10 ± 10.49**	**150.46 ± 9.24**	**157.11 ± 9.62**
MUs	2A	1051 ± 28	1067 ± 30	1123 ± 52	1056 ± 32	1089 ± 32	1134 ± 45
	3A	1051 ± 31	1075 ± 32	1146 ± 61	1065 ± 35	1109 ± 36	1168 ± 57
	4A	1046 ± 33	1071 ± 32	1147 ± 70	1061 ± 27	1109 ± 36	1196 ± 64
	4A‐HG	1077 ± 30	1106 ± 40	1217 ± 83	1141 ± 49	1200 ± 54	1277 ± 60

**FIGURE 5:**
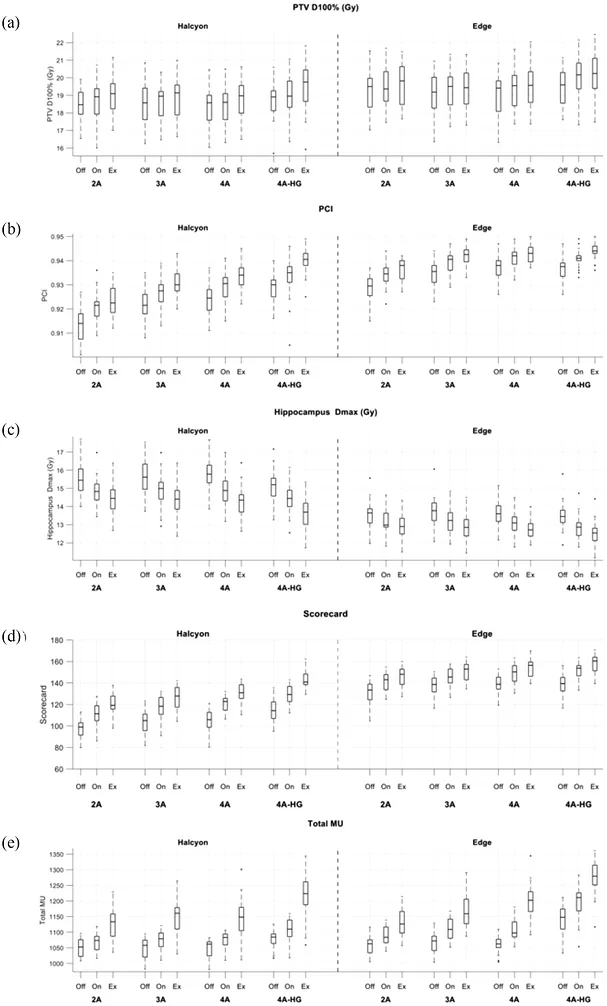
Boxplots of the selected dosimetric metrics (PTV D100%, PCI, and hippocampus Dmax), scorecard results, and MUs for plans generated using different arc configurations (2A, 3A, 4A, and 4A‐HG) and convergence modes (“Off”, “On”, and “Extended”) on Halcyon and Edge.

Selected dosimetric metrics are presented in Table 1, including PTV D100%, PCI, and hippocampus Dmax, with mean values (and standard deviation) across 20 patients for each arc configuration and convergence mode on both Halcyon and Edge. The corresponding boxplots showing the distributions of these selected dosimetric metrics are presented in Figure 5. The complete set of results and distributions for all dosimetric metrics is provided in Table S6 and Figure S2.

In terms of arc configuration, the proposed 4A‐HG resulted in superior plan quality metrics across most scenarios, with the statistical analysis results provided in Table S7. Notably, PTV D100% was significantly improved with 4A‐HG on Edge using the “On” and “Extended” convergence modes (all *p* < 0.05 compared with other arc configurations). For example, with the “On” convergence mode, mean PTV D100% across 20 patients increased from 19.433 Gy with 2A to 20.011 Gy with 4A‐HG (*p* = 0.009). The 4A‐HG configuration also significantly improved PCI, except when comparing with 4A on Edge using “Off” and “On” convergence modes (all *p* < 0.05 except for these two scenarios). The highest mean PCI was achieved using 4A‐HG with the “Extended” convergence mode (0.940 on Halcyon and 0.944 on Edge). Hippocampus Dmax was significantly reduced with 4A‐HG on Halcyon across all modes (*p* < 0.01) and on Edge for the “On” and “Extended” convergence modes (*p* < 0.05). For example, on Halcyon, the mean hippocampus Dmax decreased from 14.446 Gy to 13.662 Gy (*p* < 0.001) using the “Extended” convergence mode when changing arc configurations from 2A to 4A‐HG. A similar trend was observed on Edge, with the mean hippocampus Dmax decreasing from 12.945 Gy to 12.566 Gy (*p* = 0.003).

Analysis of convergence modes showed that more extensive optimizations were associated with significantly improved PTV and hippocampus metrics, with statistical results provided in Table S8. For example, on Halcyon using 4A‐HG, mean PTV D100% increased from 18.740 Gy to 19.416 Gy (*p* = 0.002), accompanied by improved hippocampal sparing, with mean hippocampus Dmax reduced from 15.144 Gy to 13.622 Gy (*p* < 0.001) when the convergence mode was changed from “Off” to “Extended”. Notably, the combination of 4A‐HG and the “Extended” convergence mode was the only configuration on Halcyon that enabled all plans generated using our RapidPlan model to meet the RTOG 0933 per‐protocol hippocampus Dmax constraint of ≤ 16 Gy. On Edge, similar improvements in both PTV D100% and hippocampus Dmax were also observed with 4A‐HG when the convergence mode was changed from “Off” to “Extended”, with mean PTV D100% increasing from 19.507 Gy to 20.170 Gy (*p* = 0.003) and mean hippocampus Dmax decreasing from 13.508 Gy to 12.566 Gy (*p* < 0.001).

### 3.3 Scorecard evaluation

The custom dosimetric scorecard was calculated for each plan according to Figure 3 and Table S5 to facilitate a more comprehensive evaluation of overall plan quality, with results presented in Table 1 and distributions shown in Figure 5. Statistical analyses provided in Tables S7 (arc configuration comparisons) and Table S8 (convergence mode comparisons).  Representative scorecards from an example patient are provided in Table S9 to illustrate the individual dosimetric metrics, the corresponding scores assigned to each metric, and the overall plan scores.

The scorecard results showed that 4A‐HG consistently and significantly improved plan quality on Halcyon compared with other arc configurations across all convergence modes, and on Edge when using the “On” and “Extended” convergence modes. In addition, the use of more extensive convergence modes was consistently associated with significantly higher scorecard values. Out of a maximum score of 184, the highest mean score across 20 patients was 157, achieved on Edge using 4A‐HG with the “Extended” convergence mode.

### 3.4 MUs and patient‐specific QA

Total MUs were also included in the evaluation, as presented in Table 1 and Figure 5, with statistical analyses provided in Tables S7 and S8. It was consistently observed that 4A‐HG was associated with increased MUs compared with the other arc configurations (all *p* < 0.005), and that more extensive convergence mode settings further increased MUs across all pairwise comparisons (all *p* < 0.001) on both Halcyon and Edge.

To assess whether the increased MUs affected plan deliverability and dosimetric accuracy, the five highest‐MU plans on each linac platform were selected for patient‐specific QA. The averaged gamma passing rates were 100% on Halcyon and 98.78% on Edge (field‐based averages). All fields achieved ≥ 95%, except for one field on Edge (94.6%), which still exceeded the action limit of 90%. All plans met our institutional QA criteria for clinical plans. The QA results demonstrated that the use of 4A‐HG or the “Extended” convergence mode did not affect plan deliverability, with dosimetric accuracy remaining within acceptable limits.

## 4 DISCUSSION

In this study, we proposed a 4A‐HG arc configuration for HA‐WBRT and systematically evaluated its performance relative to conventional open‐field geometries with varying numbers of arcs (2A, 3A, and 4A) on both an O‐ring linac (Halcyon) and a C‐arm linac (Edge). The dosimetric impact of convergence mode settings during optimization was also assessed. A KBP model was used to generate plans in a consistent and efficient approach, eliminating inter‐planner variability across all comparisons. In addition to the dosimetric constraints from RTOG 0933, a custom‐designed dosimetric scorecard was employed to enable a more comprehensive assessment of overall plan quality.

Our results demonstrate that the proposed 4A‐HG provides the most favorable dosimetric performance among the evaluated arc configurations, with improved target coverage and hippocampal sparing observed on both linac platforms, while increasing the number of open‐field arcs alone showed only limited improvement in plan quality. By combining two full‐field arcs with two additional arcs incorporating superior‐inferior field restriction near the hippocampal region, 4A‐HG enables more effective dose modulation adjacent to the hippocampus, reducing hippocampal dose while maintaining adequate whole‐brain coverage. Although the absolute differences in some dosimetric metrics were modest, the improvements with 4A‐HG were consistent and statistically significant across most comparisons, particularly for PCI and hippocampus Dmax. These dosimetric improvements were also reflected in the scorecard evaluation, with 4A‐HG consistently achieving the highest scores on Halcyon across all convergence modes, and on Edge when using the “On” and “Extended” modes. These findings indicate that the incorporation of partial‐field arcs improves the balance between target coverage and hippocampal sparing in HA‐WBRT VMAT planning.

The dosimetric impact of convergence mode was also found to be significant in this study, with more extensive modes consistently improving plan quality, due to increased optimization iterations and more stringent convergence criteria. Previous studies^15^, ^16^, ^17^, ^18^ have evaluated convergence modes in other treatment sites, such as breast, prostate, and head and neck; however, its impact on HA‐WBRT has not been systematically investigated. In this study, the use of more extensive modes resulted in improvements in both target coverage and OAR sparing for HA‐WBRT. Notably, the combination of 4A‐HG and the “Extended” convergence mode yielded the best overall plan quality on both linac platforms. However, the dosimetric benefits from the “Extended” convergence mode were achieved at the cost of increased optimization time, as expected, because this mode uses additional optimization iterations and more stringent convergence criteria. Based on our testing using the KBP workflow without user intervention, the planning time with the “Extended” convergence mode was approximately 243 minutes, compared with 60 minutes for “On” and 26 minutes for “Off”. Because of the GPU memory limitation in our Eclipse treatment planning system, all plans in this study were optimized and calculated using CPU rather than GPU, which further increased the planning time. In addition, the use of high‐resolution hippocampus‐related structures and a 1 mm dose calculation grid further increased the computational demand. Therefore, the actual planning time may vary depending on the available hardware, computing resources, and planning settings. Nevertheless, the trade‐off between plan quality and planning efficiency associated with convergence mode settings should be considered in clinical implementation.

An important component of this study is the use of a custom‐designed dosimetric scorecard to provide a comprehensive evaluation of plan quality. Instead of relying solely on protocol constraints, the scorecard integrates multiple dosimetric endpoints into a single quantitative framework, enabling a balanced assessment of competing planning objectives. Importantly, PTV D100% was included in our dosimetric scorecard to ensure adequate whole‐brain coverage and to reflect dose fall‐off near the hippocampus. This metric is not routinely reported in prior HA‐WBRT studies and was not included in the limited number of studies that have applied dosimetric scorecard evaluation for HA‐WBRT plan quality.^9^, ^13^ However, PTV D100% is clinically relevant, particularly in the context of hippocampal avoidance where underdosage near the avoidance region may be a concern, and it aligns with our institutional practice for clinical plan evaluation. Our scorecard evaluation also confirmed the dosimetric benefits of 4A‐HG compared with other arc configurations, as well as the use of more extensive convergence mode settings.

This study was performed on two linac platforms with different collimation systems: Halcyon with a jawless dual‐layer MLC design, and Edge with an HD‐MLC system and collimating jaws. Although inter‐platform comparison was not the primary focus of this study, Edge plans demonstrated generally lower hippocampus Dmax values and higher scores in our scorecard evaluation, reflecting improved overall plan quality, as shown in Figure 5. Previous studies have reported dosimetric performance of Halcyon and Edge in other treatment applications, such as intracranial stereotactic radiotherapy^20^; however, this has not yet been reported for HA‐WBRT. One potential explanation for the observed dosimetric differences between Halcyon and Edge plans may relate to differences in MLC design. Halcyon incorporates stacked and staggered dual‐layer MLCs with an effective 5 mm leaf width at isocenter and reduced transmission and leakage, whereas Edge utilizes the HD120 MLC system with 2.5 mm central leaf widths that may provide finer modulation capability near small and complex structures such as the hippocampus. Despite these differences, similar trends in dosimetric performance were observed, indicating that the proposed 4A‐HG and optimization strategies perform consistently across both delivery platforms, supporting broader clinical implementation of these dosimetric results.

There are several limitations to this study. First, the proposed 4A‐HG arc configuration was associated with increased MUs, indicating higher MLC modulation and increased plan complexity. However, patient‐specific QA results for the five highest‐MU plans on both linac platforms demonstrated that all evaluated plans met institutional QA criteria for clinical plans, indicating that despite the increased complexity, the plans still achieved clinically acceptable dosimetric accuracy. Second, the plans were not generated within a clinical workflow. Although our KBP model was trained on 25 high‐quality clinically approved plans, the generated plans may not represent the best achievable plan quality. In clinical practice, additional optimization or refinement may be performed to further improve the plan quality depending on clinical priorities. However, in this study, the KBP model was not intended to generate the best possible plans, but rather to ensure consistent comparisons of the dosimetric effects of different arc configurations and convergence mode settings. Finally, plan evaluation in this study did not include assessment of spatial dose distribution as performed during routine clinical review. Therefore, additional validation of the proposed 4A‐HG approach within a typical workflow is needed.

## 5 CONCLUSION

In this study, we evaluated a 4A‐HG arc configuration and the impact of convergence mode settings for HA‐WBRT using a KBP‐based planning workflow on two linac platforms with different collimation designs. The proposed 4A‐HG demonstrated improved target coverage and hippocampal sparing compared with conventional open‐field arc configurations, with consistent improvements in overall plan quality, as evaluated by a custom dosimetric scorecard, across both platforms. More extensive convergence mode settings further enhanced plan quality, at the cost of increased optimization time. Overall, these results suggest that the proposed planning strategies can improve plan quality for HA‐WBRT and support their use in clinical practice.

## AUTHOR CONTRIBUTIONS

**Anh Lam**: Investigation, Methodology, Writing—Original Draft **Kirk Luca**: Supervision, Conceptualization, Investigation, Methodology, Validation, Visualization, Writing—Original Draft, Writing—Review & Editing **Mandeep Kaur**: Investigation, Writing—Review & Editing **Keyur Shah**: Investigation, Writing—Review & Editing **Julia Seng**: Investigation, Writing—Review & Editing **Kathryn Benner**: Investigation, Writing—Review & Editing **Matthew Thomas**: Data Curation, Writing—Review & Editing **Hui‐Kuo Shu**: Writing—Review & Editing **Xiaofeng Yang**: Writing—Review & Editing **Chih‐Wei Chang**: Writing—Review & Editing **Justin Roper**: Conceptualization, Methodology, Writing—Review & Editing **Jie Ding**: Supervision, Conceptualization, Investigation, Methodology, Validation, Visualization, Formal analysis, Writing—Original Draft, Writing—Review & Editing

## CONFLICT OF INTEREST STATEMENT

The authors declare no conflicts of interest.

## ETHICS OR COMPLIANCE STATEMENT

This retrospective study was approved by the Institutional Review Board (IRB) of Emory University.

## Supporting information




**Supporting Information**: acm270771‐sup‐0001‐Figure S1‐S2.docx

**Supporting Information**: acm270771‐sup‐0002‐Table S1‐S9.docx

## Data Availability

Research data are stored in an institutional repository and may be made available from the corresponding author upon reasonable request and with appropriate approvals, in accordance with institutional policy.
