# Supplementary material for: Beam geometry optimization and convergence mode evaluation for knowledge‐based planning of hippocampal avoidance whole‐brain radiotherapy: A cross‐platform study with quantitative scorecard assessment
Source: J Appl Clin Med Phys. 2026 Aug 25;27(9):e70771. doi: 10.1002/acm2.70771 (PMC13504367; doi:10.1002/acm2.70771)
Supplement: Supplementary file 1 [file ACM2-27-e70771-s001.docx]

**Supplementary Materials – Supplementary Tables**

**Table S1.** RTOG 0933 Dose Constraints (Prescription: 30 Gy in 10 Fractions)

| Structures | Dosimetric metrics | Per protocol | Variation acceptable |
| --- | --- | --- | --- |
| PTV | D_98%_  D_2%_ | ≥ 25 Gy  ≤ 37.5 Gy | < 25Gy*  ≤ 40 Gy |
| Hippocampi | D_100%_  D_max_ | ≤ 9 Gy  ≤ 16 Gy | ≤ 10 Gy  ≤ 17 Gy |
| Optic Nerves | D_max_ | ≤ 37.5 Gy | ≤ 37.5 |
| Optic Chiasm | D_max_ | ≤ 37.5 Gy | ≤ 37.5 |

*Variation unacceptable for PTV coverage: V30Gy≤90%

**Table S2.** Optimization objectives of our RapidPlan model for HA-WBRT.

| **Structure** | **Vol [%]** | **Dose** | **Priority** | **gEUD* a** |
| --- | --- | --- | --- | --- |
| **PTV**  Upper  Upper  Upper  Lower  Lower | 0  0  3  100  100 | 105%  105%  104%  103%  103% | 220  220  220  220  220 |  |
| **PTV_Lower** **  Upper  Upper  Upper  Lower  Lower | 0  0  5  100  100 | 105%  105%  104%  103%  103% | 250  250  250  230  230 |  |
| **PTV_Upper** **  Upper  Upper  Upper  Lower  Lower | 0  0  1  100  100 | 105%  105%  104%  103%  103% | 200  200  200  200  200 |  |
| **Brainstem**  Upper | 0 | 105% | 125 |  |
| **Cochlea_L**  Line | Generated | Generated | Generated |  |
| **Cochlea_R**  Line | Generated | Generated | Generated |  |
| **Eye (R/L)**  Line | Generated | Generated | 115 |  |
| **Hippocampus PRV**  Line | Generated | Generated | 150 |  |
| **Hippocampus (L+R)**  Upper  Upper  Upper  Upper gEUD  Upper gEUD  Line | 80  50  0      Generated | Generated  Generated  Generated  35%  8.5 Gy  Generated | 165  165  180  150  150  150 | 40  4 |
| **Hippocampus_L**  Upper  Upper  Upper  Upper gEUD  Upper gEUD  Line | 80  50  0      Generated | Generated  Generated  Generated  35%  8.5 Gy  Generated | 165  165  150  150  150  150 | 40  4 |
| **Hippocampus_R**  Upper  Upper  Upper  Upper gEUD  Upper gEUD  Line | 80  50  0      Generated | Generated  Generated  Generated  35%  8.5 Gy  Generated | 165  165  150  150  150  150 | 40  4 |
| **Lens (R/L)**  Upper  Line | 0  Generated | Generated  Generated | 150  Generated |  |
| **OpticChiasm**  Upper | 0 | 105% | 150 |  |
| **OpticNerve (R/L)**  Upper  Line | 0  Generated | 105%  Generated | 140  80 |  |
| **OralCavity**  Line | Generated | Generated | Generated |  |
| **Parotid_L**  Line | Generated | Generated | Generated |  |
| **Parotid_R**  Line | Generated | Generated | Generated |  |
| **SpinalCord**  Upper | 0 | 105% | 150 |  |
| **ZAvoidFace** ***  Line | Generated | Generated | 80 |  |
| **zRing** ****  Upper  Upper gEUD | 0 | 29 Gy  23 Gy | 180  100 | 40 |

*gEUD: generalized equivalent uniform dose

**PTV_Upper is PTV superior to Hippocampus PRV and PTV_Lower is defined as PTV cropped PTV_Upper.

***zAvoidFace is defined as Boolean eyes together, expand by 5 cm twice, use the VOI box to limit expansions superiorly, crop parts extending outside of the body, crop parts extending inside PTV by 2 cm.

****zRing is defined as Extract wall of PTV by 1.1 cm outward and -0.1 cm inward then crop from inside of the Hippocampus PRV by 0.2 cm

**Table S3.** Normal tissue objective settings of our RapidPlan model for HA-WBRT.

| Priority | 200 |
| --- | --- |
| Distance from Target Border | 0.10 cm |
| Start Dose | 100% |
| End Dose | 35% |
| Fall-off | 0.12 mm^-1^ |

**Table S4.** Arc configurations used in this study.

| **Configuration** | **Number of Arcs** | **Collimator Angles** | **Aperture Restrictions** |
| --- | --- | --- | --- |
| 2A | 2 | 315°, 45° | None |
| 3A | 3 | 315°, 45°, 315° | None |
| 4A | 4 | 315°, 45°, 315°, 45° | None |
| 4A-HG | 4 | 315°, 45°, 90°, 90° | Arc 3: inferior field border limited to inferior edge of the hippocampus PTV; Arc 4: superior field border limited to superior edge of the hippocampus PTV (using MLCs on Halcyon and jaws on Edge) |

**Table S5.** Summary table listing all evaluated metrics, threshold definitions, and point values for the dosimetric scorecard. The maximum achievable total score was 184.

| **Dosimetric Metrics** | **Lower threshold (score)** | **Breakpoint (score)** | **Upper threshold (score)** |
| --- | --- | --- | --- |
| PTV D98% | 28 Gy (0) | 29 Gy (25) | 30 Gy (30) |
| PTV D2% | 34 Gy (0) | 32 Gy (25) | 31.5 Gy (30) |
| PTV D100% | 15 Gy (0) | 17 Gy (6) | 22 Gy (30) |
| PCI | 0.90 (0) | 0.92 (3) | 0.95 (10) |
| Hippocampus D100% | 10 Gy (0) | 9 Gy (10) | 7 Gy (24) |
| Hippocampus Dmax | 17 Gy (0) | 16 Gy (10) | 12 Gy (24) |
| Left Optic Nerve Dmax | 37.5 Gy (0) | 33 Gy (8) | 31.5 Gy (12) |
| Right Optic Nerve Dmax | 37.5 Gy (0) | 33 Gy (8) | 31.5 Gy (12) |
| Optic Chiasm Dmax | 37.5 Gy (0) | 33 Gy (8) | 31.5 Gy (12) |

**Table S6.** Comprehensive summary of all dosimetric metrics (based on RTOG 0933 constraints, with additional evaluation of PTV D100% and PCI), scorecard results, and total MUs for plans generated using different arc configurations (2A, 3A, 4A, and 4A-HG) and CMs (“Off”, “On”, and “Extended”) on Halcyon and TrueBeam Edge. Values are reported as mean ± standard deviation for 20 patients. Bold values indicate the best dosimetric performance among arc configurations within each CM. PTV = planning target volume; PCI = Paddick conformity index; MU = monitor unit; CM = convergence mode.

|  |  | **Halcyon** | | | **Edge** | | |
| --- | --- | --- | --- | --- | --- | --- | --- |
| Metrics | Arc configuration | CM “Off” | CM “On” | CM “Extended” | CM “Off” | CM “On” | CM “Extended” |
| PTV D98% (Gy) | 2A | 29.076 ± 0.192 | 29.124 ± 0.176 | 29.206 ± 0.171 | 29.361 ± 0.175 | 29.419 ± 0.149 | 29.451 ± 0.130 |
|  | 3A | 29.044 ± 0.162 | 29.114 ± 0.153 | 29.209 ± 0.160 | 29.344 ± 0.177 | 29.422 ± 0.147 | 29.473 ± 0.125 |
|  | 4A | 29.024 ± 0.183 | 29.112 ± 0.191 | 29.203 ± 0.172 | 29.331 ± 0.183 | 29.419 ± 0.150 | 29.474 ± 0.121 |
|  | 4A-HG | **29.133 ± 0.167** | **29.215 ± 0.195** | **29.328 ± 0.130** | **29.368 ± 0.147** | **29.462 ± 0.113** | **29.514 ± 0.103** |
| PTV D2% (Gy) | 2A | 33.375 ± 0.200 | 33.008 ± 0.173 | 32.802 ± 0.202 | 32.562 ± 0.191 | 32.275 ± 0.149 | 32.164 ± 0.164 |
|  | 3A | 33.063 ± 0.190 | 32.727 ± 0.161 | 32.494 ± 0.176 | 32.290 ± 0.174 | 32.057 ± 0.148 | 31.919 ± 0.153 |
|  | 4A | 32.964 ± 0.207 | 32.577 ± 0.179 | 32.321 ± 0.188 | **32.171 ± 0.164** | **31.944 ± 0.144** | **31.792 ± 0.146** |
|  | 4A-HG | **32.799 ± 0.198** | **32.440 ± 0.188** | **32.145 ± 0.164** | 32.315 ± 0.161 | 32.061 ± 0.127 | 31.853 ± 0.161 |
| PTV D100% (Gy) | 2A | 18.482 ± 0.906 | 18.672 ± 1.201 | 18.948 ± 1.121 | 19.206 ± 1.265 | 19.433 ± 1.224 | 19.613 ± 1.254 |
|  | 3A | 18.470 ± 1.280 | 18.668 ± 1.111 | 18.880 ± 1.232 | 19.082 ± 1.267 | 19.343 ± 1.170 | 19.402 ± 1.212 |
|  | 4A | 18.384 ± 1.230 | 18.492 ± 1.169 | 18.787 ± 1.107 | 19.005 ± 1.212 | 19.432 ± 1.174 | 19.498 ± 1.222 |
|  | 4A-HG | **18.740 ± 1.108** | **19.028 ± 1.116** | **19.416 ± 1.427** | **19.507 ± 1.022** | **20.011 ± 1.202** | **20.170 ± 1.208** |
| PCI | 2A | 0.913 ± 0.007 | 0.921 ± 0.006 | 0.923 ± 0.007 | 0.928 ± 0.006 | 0.934 ± 0.005 | 0.936 ± 0.005 |
|  | 3A | 0.921 ± 0.007 | 0.927 ± 0.007 | 0.931 ± 0.006 | 0.935 ± 0.006 | 0.939 ± 0.005 | 0.942 ± 0.004 |
|  | 4A | 0.924 ± 0.007 | 0.930 ± 0.006 | 0.934 ± 0.005 | **0.937 ± 0.005** | **0.941 ± 0.004** | 0.943 ± 0.004 |
|  | 4A-HG | **0.929 ± 0.007** | **0.933 ± 0.009** | **0.940 ± 0.005** | **0.937 ± 0.005** | **0.941 ± 0.004** | **0.944 ± 0.004** |
| Hippocampus D100% (Gy) | 2A | 8.382 ± 0.496 | 8.223 ± 0.421 | 8.068 ± 0.452 | 8.120 ± 0.348 | 7.996 ± 0.341 | 7.931 ± 0.332 |
|  | 3A | 8.475 ± 0.526 | 8.231 ± 0.483 | 8.063 ± 0.472 | **8.080 ± 0.359** | 7.964 ± 0.340 | 7.869 ± 0.341 |
|  | 4A | 8.557 ± 0.560 | 8.282 ± 0.510 | 8.074 ± 0.466 | 8.144 ± 0.339 | 7.973 ± 0.329 | 7.887 ± 0.326 |
|  | 4A-HG | **8.343 ± 0.503** | **8.081 ± 0.502** | **7.867 ± 0.491** | 8.115 ± 0.349 | **7.943 ± 0.355** | **7.832 ± 0.348** |
| Hippocampus  Dmax (Gy) | 2A | 15.545 ± 0.885 | 14.863 ± 0.815 | 14.446 ± 0.896 | 13.577 ± 0.762 | 13.157 ± 0.685 | 12.945 ± 0.661 |
|  | 3A | 15.623 ± 1.028 | 14.913 ± 1.015 | 14.392 ± 0.962 | 13.711 ± 0.870 | 13.150 ± 0.726 | 12.834 ± 0.673 |
|  | 4A | 15.769 ± 0.950 | 14.916 ± 0.885 | 14.323 ± 0.921 | 13.574 ± 0.759 | 13.054 ± 0.639 | 12.744 ± 0.521 |
|  | 4A-HG | **15.144 ± 0.942** | **14.370 ± 0.838** | **13.662 ± 0.813** | **13.508 ± 0.796** | **12.863 ± 0.690** | **12.566 ± 0.705** |
| Left Optic  Nerve Dmax (Gy) | 2A | 32.259 ± 1.319 | 32.088 ± 1.418 | 31.779 ± 1.305 | 31.464 ± 1.094 | 31.388 ± 1.295 | 31.388 ± 1.148 |
|  | 3A | 32.265 ± 1.240 | 31.939 ± 1.318 | 31.696 ± 1.364 | **31.240 ± 1.253** | 31.271 ± 1.155 | 31.177 ± 1.132 |
|  | 4A | 32.077 ± 1.344 | 31.857 ± 1.322 | 31.506 ± 1.485 | 31.352 ± 1.209 | **31.191 ± 1.221** | **31.012 ± 1.271** |
|  | 4A-HG | **32.010 ± 1.207** | **31.674 ± 1.185** | **31.522 ± 1.039** | 31.606 ± 1.333 | 31.327 ± 1.173 | 31.033 ± 1.275 |
| Right Optic  Nerve Dmax (Gy) | 2A | 32.629 ± 1.037 | 32.544 ± 0.833 | 32.400 ± 0.871 | 31.954 ± 0.845 | 31.820 ± 0.826 | 31.798 ± 0.813 |
|  | 3A | 32.429 ± 0.829 | 32.247 ± 0.778 | 32.204 ± 0.749 | 31.700 ± 0.692 | 31.574 ± 0.742 | 31.435 ± 0.861 |
|  | 4A | **32.308 ± 1.058** | 32.111 ± 0.828 | 32.101 ± 0.884 | **31.669 ± 0.802** | **31.444 ± 0.707** | **31.302 ± 0.788** |
|  | 4A-HG | 32.362 ± 0.888 | **31.904 ± 0.958** | **31.892 ± 0.851** | 31.745 ± 0.876 | 31.608 ± 0.829 | 31.424 ± 0.778 |
| Optic Chiasm  Dmax (Gy) | 2A | 33.544 ± 0.555 | 33.117 ± 0.601 | 32.992 ± 0.486 | 32.674 ± 0.452 | 32.318 ± 0.471 | 32.280 ± 0.439 |
|  | 3A | 33.121 ± 0.598 | 32.808 ± 0.666 | 32.695 ± 0.547 | 32.393 ± 0.526 | 32.158 ± 0.514 | 31.973 ± 0.492 |
|  | 4A | 33.206 ± 0.822 | 32.818 ± 0.627 | 32.675 ± 0.579 | **32.277 ± 0.428** | **32.091 ± 0.408** | **31.902 ± 0.398** |
|  | 4A-HG | **32.961 ± 0.578** | **32.676 ± 0.452** | **32.272 ± 0.601** | 32.539 ± 0.464 | 32.213 ± 0.441 | 32.057 ± 0.411 |
| Scorecard | 2A | 98.31 ± 9.63 | 110.83 ± 11.00 | 119.57 ± 11.39 | 130.94 ± 11.17 | 140.90 ± 9.53 | 145.36 ± 9.68 |
|  | 3A | 103.17 ± 12.39 | 116.87 ± 11.63 | 126.41 ± 11.23 | 136.30 ± 11.13 | 145.65 ± 9.10 | 150.86 ± 9.45 |
|  | 4A | 103.65 ± 12.65 | 118.61 ± 10.59 | 129.40 ± 10.68 | 138.08 ± 11.17 | 148.52 ± 8.88 | 153.87 ± 8.55 |
|  | 4A-HG | **114.32 ± 12.08** | **128.90 ± 9.24** | **142.34 ± 10.55** | **138.10 ± 10.49** | **150.46 ± 9.24** | **157.11 ± 9.62** |
| MUs | 2A | 1051 ± 28 | 1067 ± 30 | 1123 ± 52 | 1056 ± 32 | 1089 ± 32 | 1134 ± 45 |
|  | 3A | 1051 ± 31 | 1075 ± 32 | 1146 ± 61 | 1065 ± 35 | 1109 ± 36 | 1168 ± 57 |
|  | 4A | 1046 ± 33 | 1071 ± 32 | 1147 ± 70 | 1061 ± 27 | 1109 ± 36 | 1196 ± 64 |
|  | 4A-HG | 1077 ± 30 | 1106 ± 40 | 1217 ± 83 | 1141 ± 49 | 1200 ± 54 | 1277 ± 60 |

**Table S7.** Holm-adjusted *p*-values for pairwise comparisons of arc configurations (2A, 3A, 4A, and 4A-HG) on Halcyon and TrueBeam Edge, evaluated separately for each CM (“Off”, “On”, and “Extended”). Statistical significance across arc configurations was assessed using the Friedman test for repeated measures, followed by post hoc Wilcoxon signed-rank tests with Holm correction for multiple comparisons. Blank cells indicate comparisons for which the Friedman test was not significant or that did not remain significant after Holm adjustment. P-values < 0.001 are reported as “<0.001”. Comparisons involving 4A-HG versus other arc configurations are shown in bold. MU= monitor unit; CM= convergence mode; PTV = planning target volume; PCI = Paddick conformity index.

|  |  | **Halcyon** | | | | | | **Edge** | | | | | |
| --- | --- | --- | --- | --- | --- | --- | --- | --- | --- | --- | --- | --- | --- |
| Metric | CM | 2A vs 3A | 2A vs 4A | **2A vs 4A-HG** | 3A vs 4A | **3A vs 4A-HG** | **4A vs 4A-HG** | 2A vs 3A | 2A vs 4A | **2A vs 4A-HG** | 3A vs 4A | **3A vs 4A-HG** | **4A vs 4A-HG** |
| PTV D98% | Off |  | 0.013 | 0.022 |  | 0.003 | <0.001 | 0.012 | 0.005 |  |  |  | 0.042 |
|  | On |  |  | 0.006 |  | 0.007 | 0.007 |  |  | 0.006 |  | 0.005 | 0.006 |
|  | Extended |  |  | <0.001 |  | <0.001 | <0.001 | 0.004 | 0.004 | <0.001 |  | <0.001 | <0.001 |
| PTV D2% | Off | <0.001 | <0.001 | <0.001 | 0.010 | <0.001 | <0.001 | <0.001 | <0.001 | <0.001 | <0.001 |  | <0.001 |
|  | On | <0.001 | <0.001 | <0.001 | <0.001 | <0.001 | <0.001 | <0.001 | <0.001 | <0.001 | <0.001 |  | <0.001 |
|  | Extended | <0.001 | <0.001 | <0.001 | <0.001 | <0.001 | <0.001 | <0.001 | <0.001 | <0.001 | <0.001 | 0.002 | 0.003 |
| PTV D100% | Off |  |  |  |  |  |  |  |  |  |  |  |  |
|  | On |  |  |  |  |  |  |  |  | 0.009 |  | 0.007 | 0.009 |
|  | Extended |  |  |  |  |  | 0.010 |  |  | 0.011 |  | 0.002 | 0.002 |
| PCI | Off | <0.001 | <0.001 | <0.001 | 0.005 | <0.001 | <0.001 | <0.001 | <0.001 | <0.001 | <0.001 | <0.001 |  |
|  | On | <0.001 | <0.001 | <0.001 | 0.001 | 0.003 | 0.003 | <0.001 | <0.001 | <0.001 | <0.001 | 0.005 |  |
|  | Extended | <0.001 | <0.001 | <0.001 | <0.001 | <0.001 | <0.001 | <0.001 | <0.001 | <0.001 | 0.003 | <0.001 | 0.015 |
| Hippocampus D100% | Off |  | 0.010 |  |  |  | 0.002 |  |  |  |  |  |  |
|  | On |  |  | 0.040 |  | 0.018 | <0.001 |  |  |  |  |  |  |
|  | Extended |  |  | 0.001 |  | 0.003 | 0.001 | 0.003 | 0.041 | 0.003 |  |  | 0.029 |
| Hippocampus Dmax | Off |  |  | 0.005 |  | 0.005 | <0.001 |  |  |  |  | 0.046 |  |
|  | On |  |  | <0.001 |  | <0.001 | <0.001 |  |  | 0.003 |  | 0.002 | 0.024 |
|  | Extended |  |  | <0.001 |  | <0.001 | <0.001 | 0.024 | 0.010 | 0.003 | 0.037 | 0.003 | 0.037 |
| Left Optic Nerve Dmax | Off |  |  |  |  |  |  |  |  |  |  |  |  |
|  | On |  |  |  |  |  |  |  |  |  |  |  |  |
|  | Extended |  |  |  |  |  |  |  | 0.015 | 0.045 |  |  |  |
| Right Optic Nerve Dmax | Off |  |  |  |  |  |  |  |  |  |  |  |  |
|  | On |  | 0.023 | 0.002 |  |  |  |  | 0.031 |  |  |  |  |
|  | Extended |  |  | 0.012 |  |  |  | 0.005 | 0.001 | 0.003 |  |  |  |
| Optic Chiasm Dmax | Off | 0.005 | 0.045 | 0.001 |  |  |  | 0.005 | 0.001 |  |  |  | 0.004 |
|  | On | 0.009 | 0.011 | 0.002 |  |  |  |  | 0.002 |  |  |  |  |
|  | Extended | 0.003 | 0.002 | <0.001 |  | 0.001 | 0.002 | 0.001 | <0.001 |  |  |  | 0.044 |
| Scorecard | Off | 0.010 | 0.005 | <0.001 |  | <0.001 | <0.001 | <0.001 | <0.001 | <0.001 | 0.015 |  |  |
|  | On | 0.001 | <0.001 | <0.001 |  | <0.001 | <0.001 | <0.001 | <0.001 | <0.001 | <0.001 | <0.001 | 0.019 |
|  | Extended | <0.001 | <0.001 | <0.001 | 0.019 | <0.001 | <0.001 | <0.001 | <0.001 | <0.001 | 0.002 | <0.001 | 0.005 |
| MU | Off |  |  | 0.001 |  | 0.001 | <0.001 | 0.001 |  | <0.001 |  | <0.001 | <0.001 |
|  | On |  |  | <0.001 |  | <0.001 | <0.001 | <0.001 | <0.001 | <0.001 |  | <0.001 | <0.001 |
|  | Extended |  |  | <0.001 |  | 0.001 | 0.002 | <0.001 | <0.001 | <0.001 | <0.001 | <0.001 | <0.001 |

**Table S8.** Holm-adjusted *p*-values for pairwise comparisons of CMs (“Off”, “On”, and “Extended”) on Halcyon and TrueBeam Edge, evaluated separately for each arc configuration (2A, 3A, 4A, and 4A-HG). Statistical significance across arc configurations was assessed using the Friedman test for repeated measures, followed by post hoc Wilcoxon signed-rank tests with Holm correction for multiple comparisons. Blank cells indicate comparisons for which the Friedman test was not significant or that did not remain significant after Holm adjustment. P-values < 0.001 are reported as “<0.001”. MU= monitor unit; CM= convergence mode; PTV = planning target volume; PCI = Paddick conformity index.

|  |  | **Halcyon** | | | **TrueBeam Edge** | | |
| --- | --- | --- | --- | --- | --- | --- | --- |
| Metrics | Arc configuration | CM:  Off vs On | CM:  Off vs Extended | CM:  On vs Extended | CM:  Off vs On | CM:  Off vs Extended | CM:  On vs Extended |
| PTV D98% | 2A | 0.004 | <0.001 | <0.001 | <0.001 | <0.001 | <0.001 |
|  | 3A | <0.001 | <0.001 | <0.001 | <0.001 | <0.001 | <0.001 |
|  | 4A | <0.001 | <0.001 | <0.001 | <0.001 | <0.001 | <0.001 |
|  | 4A-HG | <0.001 | <0.001 | <0.001 | <0.001 | <0.001 | <0.001 |
| PTV D2% | 2A | <0.001 | <0.001 | <0.001 | <0.001 | <0.001 | <0.001 |
|  | 3A | <0.001 | <0.001 | <0.001 | <0.001 | <0.001 | <0.001 |
|  | 4A | <0.001 | <0.001 | <0.001 | <0.001 | <0.001 | <0.001 |
|  | 4A-HG | <0.001 | <0.001 | <0.001 | <0.001 | <0.001 | <0.001 |
| PTV D100% | 2A |  | 0.014 | 0.037 | 0.037 | 0.007 | 0.037 |
|  | 3A |  | 0.027 |  | 0.027 | 0.027 |  |
|  | 4A |  | 0.017 | 0.017 |  |  |  |
|  | 4A-HG | 0.018 | 0.002 | 0.018 | 0.004 | 0.003 |  |
| PCI | 2A | <0.001 | <0.001 | 0.026 | <0.001 | <0.001 | 0.003 |
|  | 3A | <0.001 | <0.001 | <0.001 | <0.001 | <0.001 | <0.001 |
|  | 4A | <0.001 | <0.001 | <0.001 | <0.001 | <0.001 | <0.001 |
|  | 4A-HG | 0.001 | <0.001 | <0.001 | <0.001 | <0.001 | <0.001 |
| Hippocampus D100% | 2A | <0.001 | <0.001 | <0.001 | <0.001 | <0.001 | <0.001 |
|  | 3A | <0.001 | <0.001 | <0.001 | <0.001 | <0.001 | <0.001 |
|  | 4A | <0.001 | <0.001 | <0.001 | <0.001 | <0.001 | <0.001 |
|  | 4A-HG | <0.001 | <0.001 | <0.001 | <0.001 | <0.001 | <0.001 |
| Hippocampus Dmax | 2A | <0.001 | <0.001 | <0.001 | <0.001 | <0.001 | <0.001 |
|  | 3A | <0.001 | <0.001 | <0.001 | <0.001 | <0.001 | <0.001 |
|  | 4A | <0.001 | <0.001 | <0.001 | <0.001 | <0.001 | <0.001 |
|  | 4A-HG | <0.001 | <0.001 | <0.001 | <0.001 | <0.001 | <0.001 |
| Left Optic  Nerve Dmax | 2A |  |  |  |  |  |  |
|  | 3A |  | 0.002 |  |  |  |  |
|  | 4A |  | 0.012 | 0.034 | 0.039 | 0.030 | 0.039 |
|  | 4A-HG |  | 0.007 |  | 0.044 | 0.003 | 0.018 |
| Right Optic  Nerve Dmax | 2A |  |  |  |  |  |  |
|  | 3A |  |  |  |  |  |  |
|  | 4A |  |  |  | 0.022 | 0.002 |  |
|  | 4A-HG | 0.002 | 0.010 |  |  | 0.003 |  |
| Optic Chiasm  Dmax | 2A | 0.002 | 0.002 |  | <0.001 | <0.001 |  |
|  | 3A | 0.001 | 0.001 |  | 0.022 | 0.001 | 0.022 |
|  | 4A | 0.002 | 0.001 |  | 0.001 | <0.001 | <0.001 |
|  | 4A-HG | 0.004 | <0.001 | <0.001 | <0.001 | <0.001 | 0.008 |
| Scorecard | 2A | <0.001 | <0.001 | <0.001 | <0.001 | <0.001 | <0.001 |
|  | 3A | <0.001 | <0.001 | <0.001 | <0.001 | <0.001 | <0.001 |
|  | 4A | <0.001 | <0.001 | <0.001 | <0.001 | <0.001 | <0.001 |
|  | 4A-HG | <0.001 | <0.001 | <0.001 | <0.001 | <0.001 | <0.001 |
| MUs | 2A | <0.001 | <0.001 | <0.001 | <0.001 | <0.001 | <0.001 |
|  | 3A | <0.001 | <0.001 | <0.001 | <0.001 | <0.001 | <0.001 |
|  | 4A | <0.001 | <0.001 | <0.001 | <0.001 | <0.001 | <0.001 |
|  | 4A-HG | <0.001 | <0.001 | <0.001 | <0.001 | <0.001 | <0.001 |

**Table S9.** Representative scorecards for six plans from the same example patient, including the dosimetric values, corresponding individual metric scores, and overall scores. For each linac platform, the selected plans illustrate the dosimetric effects of the proposed 4A-HG beam geometry (compared with 2A) and the convergence mode (“Off” vs “Extended”).

|  | **Halcyon** | | | | | | **Edge** | | | | | |
| --- | --- | --- | --- | --- | --- | --- | --- | --- | --- | --- | --- | --- |
| **Metric** | **2A-Off** | **Score** | **4A-HG-Off** | **Score** | **4A-HG-Extended** | **Score** | **2A-Off** | **Score** | **4A-HG-Off** | **Score** | **4A-HG-Extended** | **Score** |
| PTV D98% (Gy) | 29.12 | **25.59** | 29.28 | **26.39** | 29.42 | **27.08** | 29.47 | **27.36** | 29.47 | **27.34** | 29.58 | **27.88** |
| PTV D2% (Gy) | 33.11 | **11.16** | 32.46 | **19.23** | 31.88 | **26.22** | 32.47 | **19.19** | 32.12 | **23.54** | 31.67 | **28.35** |
| PTV D100% (Gy) | 17.04 | **6.17** | 18.38 | **12.61** | 18.56 | **13.48** | 19.97 | **20.23** | 20.28 | **21.76** | 20.95 | **24.96** |
| PCI | 0.92 | **3.47** | 0.94 | **7.67** | 0.95 | **9.07** | 0.94 | **6.97** | 0.94 | **8.60** | 0.95 | **9.77** |
| Hippocampus Dmax (Gy) | 15.79 | **10.74** | 14.69 | **14.57** | 13.61 | **18.37** | 13.42 | **19.05** | 13.38 | **19.17** | 12.12 | **23.59** |
| Hippocampus D100% (Gy) | 8.26 | **15.19** | 8.16 | **15.89** | 7.75 | **18.74** | 8.09 | **16.34** | 8.10 | **16.34** | 7.76 | **18.69** |
| OpticChiasm Dmax (Gy) | 32.71 | **8.76** | 31.30 | **12.00** | 31.25 | **12.00** | 31.70 | **11.46** | 31.67 | **11.54** | 31.56 | **11.84** |
| OpticNerve L Dmax (Gy) | 32.84 | **8.43** | 32.60 | **9.07** | 31.43 | **12.00** | 32.35 | **9.74** | 32.08 | **10.44** | 31.05 | **12.00** |
| OpticNerve R Dmax (Gy) | 33.28 | **7.50** | 32.07 | **10.49** | 31.68 | **11.53** | 32.01 | **10.64** | 32.05 | **10.53** | 31.69 | **11.51** |
| **Total score** | **-** | **97.02** | **-** | **127.92** | **-** | **148.49** | **-** | **140.97** | **-** | **149.25** | **-** | **168.59** |
