# Supplementary material for: Beam geometry optimization and convergence mode evaluation for knowledge‐based planning of hippocampal avoidance whole‐brain radiotherapy: A cross‐platform study with quantitative scorecard assessment
Source: J Appl Clin Med Phys. 2026 Aug 25;27(9):e70771. doi: 10.1002/acm2.70771 (PMC13504367; doi:10.1002/acm2.70771)
Supplement: Supplementary file 2 [file ACM2-27-e70771-s002.docx]

**Supplementary Materials – Supplementary Figures**

**Figure S1.** BEV comparison for two machine configurations, PTV is segmented in red. (A) Varian Halcyon. (B) Varian Edge.

**Figure S2.** Boxplots of all dosimetric metrics (based on RTOG 0933 constraints, with additional evaluation of PTV D100% and PCI), scorecard results, and total MUs for plans generated using different arc configurations (2A, 3A, 4A, and 4A-HG) and CMs (“Off”, “On”, and “Extended”) on Halcyon and TrueBeam Edge. PTV = planning target volume; PCI = Paddick conformity index; MU = monitor unit; CM = convergence mode.

(A)

(B)

(C)

(D)

(F)

(E)

(G)

(H)

(I)

(J)

(K)
